# Ultrathin Multilayer Textile Structure with Enhanced EMI Shielding and Air-Permeable Properties

**DOI:** 10.3390/polym13234176

**Published:** 2021-11-29

**Authors:** Shi Hu, Dan Wang, Aravin Prince Periyasamy, Dana Kremenakova, Jiri Militky, Maros Tunak

**Affiliations:** 1 Department of Material Engineering, Faculty of Textile Engineering, Technical University of Liberec, Studenska, 1402/2, 46117 Liberec, Czech Republic; dan.wang@tul.cz (D.W.); aravinprince@gmail.com (A.P.P.); dana.kremenakova@tul.cz (D.K.); jiri.militky@tul.cz (J.M.); 2 Department of Bioproducts and Biosystems, School of Chemical Engineering, Aalto University, 02150 Espoo, Finland; 3 Department of Textile Evaluation, Faculty of Textile Engineering, Technical University of Liberec, Studenska, 1402/2, 46117 Liberec, Czech Republic; maros.tunak@tul.cz

**Keywords:** electromagnetic shielding effectiveness, multilayer structure, porosity, air permeability

## Abstract

A textile material’s electromagnetic interference (EMI) shielding effectiveness mainly depends on the material’s electrical conductivity and porosity. Enhancing the conductivity of the material surface can effectively improve the electromagnetic shielding effectiveness. However, the use of highly conductive materials increases production cost, and limits the enhancement of electromagnetic shielding effectiveness. This work aims to improve the EMI shielding effectiveness (EMSE) by using an ultrathin multilayer structure and the air-permeable textile MEFTEX. MEFTEX is a copper-coated non-woven ultrathin fabric. The single-layer MEFTEX SE test results show that the higher its mass per unit area (MEFTEX 30), the better its SE property between 56.14 dB and 62.53 dB in the frequency band 30 MHz–1.5 GHz. Through comparative testing of three groups samples, a higher electromagnetic shielding effect is obtained via multilayer structures due to the increase in thickness and decrease of volume electrical resistivity. Compared to a single layer, the EMI shielding effectiveness of five layers of MEFTEX increases by 44.27–83.8%. Due to its ultrathin and porous structure, and considering the balance from porosity and SE, MEFTEX 10 with three to four layers can still maintain air permeability from 2942 L/m^2^/s–3658 L/m^2^/s.

## 1. Introduction

The influence of electromagnetic waves on human health is contentious, but to date, scientific consensus has been that excessive electromagnetic radiation impacts human health [1,2]. In addition, radiation due to electromagnetic effects causes unnecessary electromagnetic interference [3]. The electric field in radiation can interact with the electrons in the metal conductors, and this interference can cause some sensitive electronics to malfunction [4]. To prevent the extra electromagnetic wave, due to the electrostatic equipotentiality of metals, electromagnetic interference (EMI) shielding material can effectively shield external electric fields from electromagnetic interference. The setup of EMI shielding protective clothing should comply with the minimum health and safety requirements regarding the exposure of workers to the risks arising from physical agents (18th individual directive within the meaning of Article 16(1) of Directive 89/391/EEC), which require the employee to use suitable protective equipment to work in high electromagnetic radiation environments [5]. The research on metal-coated textiles has proven the effectiveness of EMI shielding in many applications. Compared to other EMI shielding materials like metal, carbon-polymer composites, and nanofibrils, conductive textiles not only provide effective EMI shielding due to the textile-based structure of metal-coated textile material, but also have good wearable properties, such as air permeability and thermal properties [6]. 

Three phenomena determine how electromagnetic field strength is lost as it interacts the shielding objects. These phenomena are absorption, attenuation due to reflection, and attenuation due to internal reflection [7]. The primary mechanisms of EMI shielding material are reflection and absorption [8]. The loss due to the multiple reflections usually can be ignored due to the distance between the reflecting surfaces, or the interface is large compared to the skin depth [9]. When a textile material has a low thickness, the primary mechanism for EMI shielding metal-coated textiles is reflection. In this case, to enhance the EMI shielding effectiveness (SE) of textile shielding clothing, the most common method is coating the low electrical resistance metals to enhance the material conductivity, for example, by using silver or gold as coating metal. This concept is not widely implemented, which may be because these metals are too expensive for large-scale industrial production and the consumer market, especially for technical clothing use [10,11]. The MXene/metal oxides nanostructured coating for textile EMI shielding is being quickly developed [12]. Regarding nano-sized metal or heavy metal coated textile, the human body’s health risks are still of concern [13]. 

As well as metal conductivity, the porosity and thickness of EMI shielding textiles significantly influence electromagnetic shielding effectiveness. The test results show that, by using aluminum foils with pores after increasing the pore size from 1 mm to 3 mm, the SE reduced by 20–37.5%. Similar results were observed from different pore sizes of EMI shielding textiles [14]. Considering the influence of thickness after increasing the fabrics’ thicknesses by using the same material, and the SE improved by 5–10 dB for each increased fabric on different frequencies [3]. Researcher S. Palanisamy studied the influence factor of textile SE. From the findings of the design of experiment (DoE) screening design, the main influence factors are the thickness of the materials, the apertures, and the strips laid angle, which have a statistically significant effect on electromagnetic shielding effectiveness [15].

Generally, compared to other EMI shielding materials such as nanofibrous membranes, porous light fabric with high SE shows that there is promise for technical textiles to achieve the balance of high air permeability and high SE with low thickness. For this reason, a particular type of composite nonwoven polyester fabric MILIFE^®^ with a surface covering by a particulate-based copper layer produced by Bochemie Ltd., Bohumin, Czech Republic, named MEFTEX^®^ used in this research. 

The purpose of this study is to investigate if the EMI shielding fabrics by using multilayer combination and mutually aligned with each other, fulfills the expectation of increased SE in the frequency band from 30 MHz to 1.5 GHz, which is specified in ASTM D 4935-10 [16], simultaneously ensure a certain degree of air permeability.

## 2. Materials and Methods

### 2.1. Material Geometric and Properties

The copper-coated nonwoven polyester fabric, named MEFTEX, is purchased from Bochemie Ltd., Bohumin, Czech Republic. The greige fabrics are nonwoven polyester-based fabrics (commercial name MILIFE) from JX Nippon ANCI, Tokyo, Japan. MILIFE is composed of a dense net of monofilaments bonded by solid spots from locally melted filaments, making it a potentially useful material for surface metallization. Three different mass per unit area (GSM; w) [g/m^2^] of greige MILIFE fabric were used for copper coating, 10 g/m^2^, 20 g/m^2^, and 30 g/m^2^. The fabric mass per unit area [g/m^2^] was measured using the standard ASTM D 377633 [17] and the sample size was 600 cm^2^. Fabric thickness (t) was measured using a thickness gauge [mm], as per standard ASTM D 5729 [18] (nonwoven samples), and pressure of 1 kPa was used. A total of 10 times measurements were performed; the mean values and 95% confidence intervals for the means are summarized in Table 1.

### 2.2. Metal Coating

**Figure 1:**
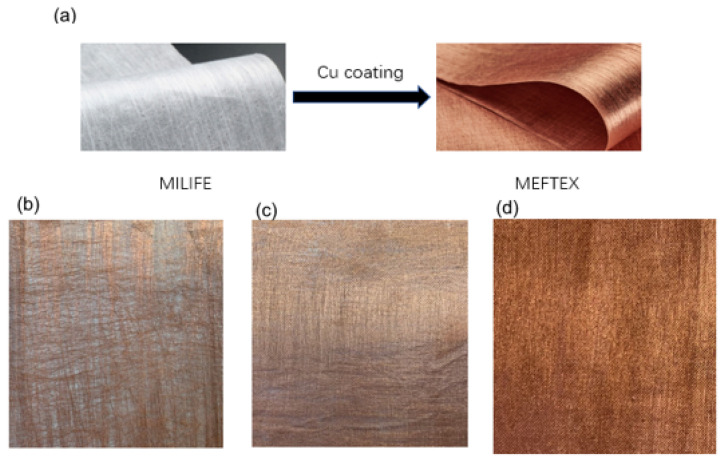
Sample appearance image (**a**) Copper coated MEFTEX (**b**) MEFTEX 10 (20 cm × 20 cm) (**c**) MEFTEX 20 (20 cm × 20 cm) (**d**) MEFTEX 30 (20 cm × 20 cm).

MEFTEX is produced via a unique roll-to-roll technological process (patent pending) based on subsequent chemical and electroplating processes. The surface activation process was carried out using an activation solution (CATAPOSIT44, supplied by Rohm and Hass company, Hoek, The Netherlands) at 45 °C for 5 min, then immersed in 10% hydrochloric acid for 1 min. Pre-activated MILIFE fabric is passing through a bath containing salt CuSO_4_ and copper nanoparticles on fiber surface obtained by the action of reducing bath (based on borohydride). By this process, a fragile metal particles dense layer (copper) was created on MILIFE fabrics’ surface (see Figure 1).

### 2.3. Characterization

#### 2.3.1. SEM and EDX

The surface morphology of the MEFTEX fabric was observed under the scanning electron microscopy (SEM) (VEGA TESCAN Inc. Brno, Czech Republic) at 20 kV. The cross-section and the surface of the Cu-coated fabric were observed. Elemental analysis was performed on the metal composition on the surface of MEFTEX (energy-dispersive X-ray spectroscopy-EDX) using an Oxford Instruments analyzer and AZTEC software Version 3.2.

#### 2.3.2. EMSE Measurements

**Figure 2:**
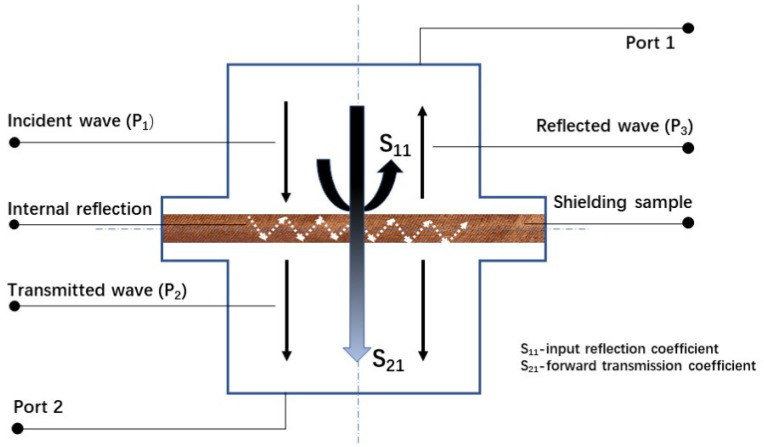
Measurement of SE for MEFTEX.

The effectiveness of the electromagnetic shielding of textiles was evaluated by the insertion loss method according to ASTM 4935-10, which is designed for the evaluation of flat materials. This standard assumes a plane wave impacts on a shielding barrier in the near zone of the electromagnetic field at a frequency of 30 MHz to 1.5 GHz. The measuring fixture consisted of a coaxial specimen holder (manufactured by Electro-Metrics, Inc., model EM-2107A), whose input and output were connected to a perimeter analyzer. A Rhode & Schwarz ZNC3 circuit analyzer was used to generate and receive an electromagnetic signal. The test principle of this method is illustrated in Figure 2. The *SE* can be interpreted by the forward transmission coefficient *S21*, which is the ratio of power without and with shielding material; the calculation method is as Equation (1):

(1) $$SE(S21)\;=\;-10log\frac{P_{1}}{P_{2}}\;=\;10log\frac{P_{2}}{P_{1}}$$

P1 is the received power without the fabric present, and P2 is the received power with the fabric present. To interpret Equation (1), by the sign “-”, the higher the value of SE, the smaller the number should be that is preceded by “-.” To perform a straightforward inference, in the following, the result of the SE value is presented without the sign “-” [19]. The electromagnetic wave reflection coefficient interpreting the electromagnetic wave signal from the transmitting antenna is reflected by the sample and received by Port 1. The ratio of the receiving reflected power (*P*_3_) and the received power without the fabric present (*P*_1_) calculates the input reflection coefficient by Equation (2):

(2) $$S11\;=\;10log\frac{P_{3}}{P_{1}}$$

The measurements were performed under the following laboratory conditions: T = 23.9 °C ± 2 °C, RH = 48% ± 5%. Each sample was measured five times at different locations.

#### 2.3.3. Volume Resistivity Test

The volume resistivity $\rho_{v}$ [Ω mm] of three groups of the sample was calculated according to standard ASTM D257-14 [20] at a temperature 23.2 ± 2 °C and a relative humidity RH of 50.7% ± 5%. The electrodes were connected with a 100 V direct current (DC) power supply. The sample was placed in an air-conditioned room for 24 h before testing. Volume resistivity $\rho_{v}$ [Ω mm] was then calculated from Equation (3):

(3) $$\rho_{v}\;=\;R_{v}\left(\frac{S}{t}\right)$$

*R_v_* is the reading of volume resistance measurement, *t* [m] is the material thickness and *S* [m^2^] is the surface area of measurement electrodes.

#### 2.3.4. Porosity (Optical) Analysis and Air Permeability

**Figure 3:**
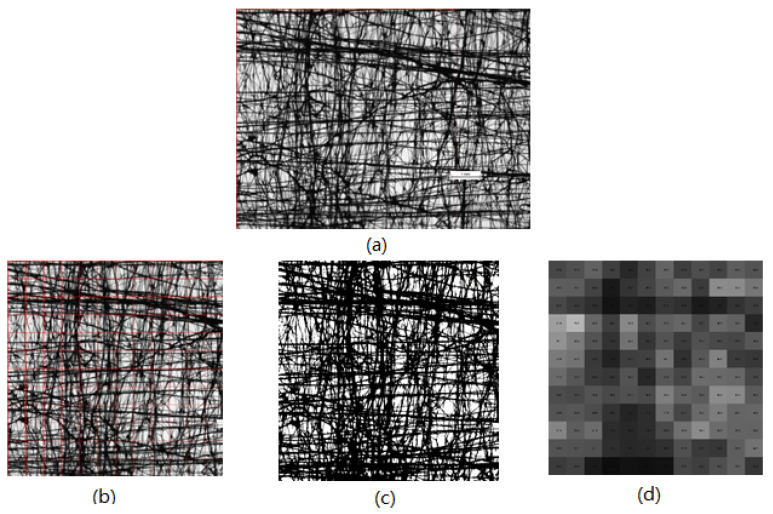
(**a**) Original image of sample MEFTEX 10 (1200 × 1600 px; 7.69 mm × 10.26 mm), (**b**) image crop and sub windows of size 100 × 100 px (0.64 mm × 0.64 mm), (**c**) binary image (**d**) map of porosity.

A fabric’s porosity has a strong positive correlation with its air permeability, which is one of the essential wearing comforts for evaluating fabrics. One characteristic of the distribution of a nonwoven fabric’s porosity is unevenness. In image analysis, a nonwoven fabric’s porosity can be expressed by the optical porosity, which is a percentage value calculated from the aperture area of the observation part divided by area of the observation part. For the porosity investigation, images of MEFTEX were obtained using a Nikon Eclipse E200 microscope in transmitted light. Images were captured as RGB image matrices of size 1200 × 1600 px. All images were captured using the same microscope. Following image analysis, for better visual representation, the original picture was cropped to 1200 × 1200 px after adjusting the intensity to size 100 × 100 px (0.64 × 0.64 mm), converting this image into a binary image. Finally, by using the MATLAB program processing this image, each grid porosity of 12 × 12 mesh structured image was generated (see Figure 3). The numbers in each grid represent the porosity, and porosity distribution can be expressed via box plot and observed via the whole generated image.

An essential factor in the comfort of fabric is air permeability (AP), which is influenced by its porosity. An AP tester (FX 3300, TEXTEST Instruments, Hertogenbosch, The Netherlands) was used at 200 Pa to conduct the AP test according to the ISO 9237 standard [21]. The AP was measured under the laboratory conditions T = 21.3 °C ± 2 °C, RH = 50% ± 5%. To test air permeability, each of three different samples was measured ten times.

## 3. Results and Discussion

### 3.1. Morphology Analysis and Elemental Analysis

**Figure 4:**
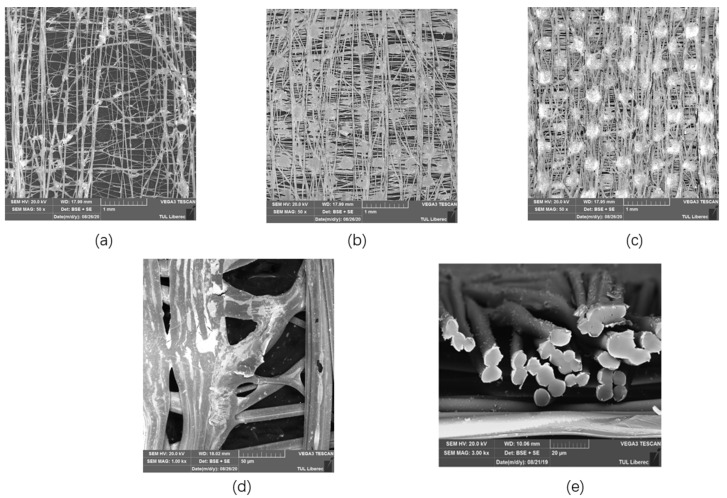
Scanning electron micrographs images of (**a**) MEFTEX 10 (50×) (**b**) MEFTEX 20 (50×) and (**c**) MEFTEX 30 (50×) (**d**) Meftex surface coated with copper micro view (**e**) A cross section view of MEFTEX.

The structure of single-layer MEFTEX 10, MEFTEX 20, and MEFTEX 30 was observed from SEM images (Figure 4). From the image, it is clear the porous structure and critical point of the nonwoven fabric MILIFE. MILIFE is composed of a dense net of monofilaments from which critical points produce locally melted filaments, making it a promising material for surface metallization. To highlight the unique processing technology of MILIFE, the porous structure is presented in the image. The porous structure decreases with the increasing mass per unit area of MILIFE nonwoven fabrics. MEFTEX 20 and MEFTEX 30′s arrangement of fibers are dense in the same area, while MEFTEX 10 has more overlapping fibers. This is the reason MEFTEX 20 and MEFTEX 30 have higher thickness and areal density. Figure 4d,e shows the surface morphologies of MEFTEX. It is apparent from the micrographs that a thin copper layer can be clearly distinguished on the MEFTEX surface.

**Figure 5:**
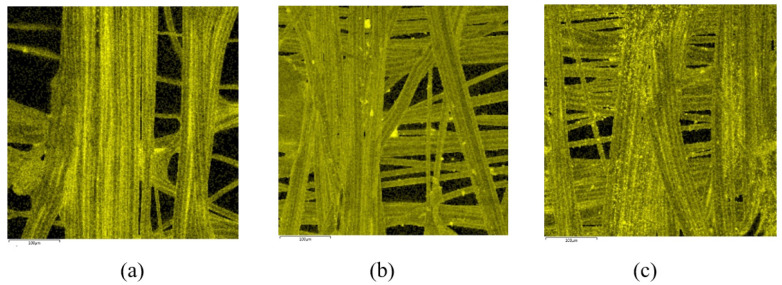
X-ray spectroscopy–EDX analysis of (**a**) MEFTEX 10 (**b**) MEFTEX 20 (**c**) MEFTEX 30 element Cu distribution.

Figure 5 displays the EDX analysis results of Cu deposition elements in the area of 450 μm × 450 μm. Five times measurement were applied; Table 2 displays the mean value and standard deviation for the detected elements. The high proportion of copper with high surface density is visible on the surface of each sample. In addition to Cu, oxygen (O), carbon (C), calcium (Ca), and titanium (Ti) are visible, and their concentration is presented in Figure 5. The presence of titanium is due to the matting of polyester by TiO_2_ before creating a MILIFE structure [22]. It is observed that the copper coating is not continuous, but distances between coated parts are over the percolation threshold. The electromagnetic SE, in particular, is extraordinarily high.

### 3.2. Single Layer EMSE of MEFTEX

**Figure 6:**
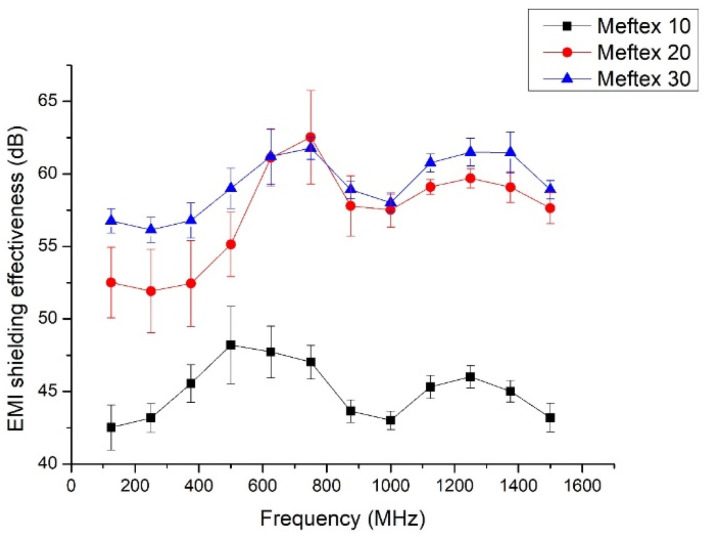
MEFTEX EMI shielding effectiveness in the frequency band from 30 MHz–1.5 GHz.

The results of single-layer MEFTEX’s EMI shielding effectiveness are displayed in Figure 6. Figure 6 shows that the MEFTEX 10 reported the lowest SE from 42.5 dB to 48.2 dB compared to other samples in the frequency band 30 MHz–1.5 GHz. MEFTEX 30 provides higher SE between 56.14 dB and 62.53 dB in the frequency band 30 MHz–1.5 GHz. According to the results, the SE of MEFTEX with a higher mass per unit area of 30 g/m^2^ (sample MEFTEX 30) performs better EMI shielding due to a larger amount of metalized fibers than MEFTEX with a basis weight of 10 g/m^2^ or 20 g/m^2^ (samples MEFTEX 10, MEFTEX 20). All samples of single-layer MEFTEX, according to the classification, evaluated in the “Excellent” category for Class II general use. For Class I Professional use, MEFTEX 10 fulfills the SE requirements of grade “AAA” (Good), while MEFTEX 20 and MEFTEX 30 fulfill the SE requirements for between a “AAAA” (Very good) and “AAAAA” (Excellent) grade [23] (see Table A1).

For conductor plate shielding materials, *SE* can be calculated by the Schelkunoff equation (Equation (4)) based on the transmission theory, with *SE_A_* [dB] being the absorbing loss of the shielding materials, *SE_R_* [dB] the single reflection loss on the surface of the shielding materials, and *SE_M_* [dB] the multi-reflection loss inside the shielding materials [24].

(4) $$SE\;=\;SE_{A}+SE_{R}+SE_{M}$$

The electromagnetic shielding efficiency of an element is characterized by its electric conductivity, permittivity, permeability, parameters of source, and properties of the ambient surroundings [9]. For EMI shielding materials with an *SE_A_* of more than 6 dB, the multi-reflection loss inside the shielding material can be ignored. When *SE_A_* is less than 10 dB, the correction term *SE_M_* should be considered. The multiple reflection correction term can be calculated via the following equation [25,26]:

(5) SEM=20log10(1−(C−1)2(C+1)210−SEA10)

where *C* = Z_S_/Z_H_, Z_s_ is the shield impedance and Z_H_ is the impedance of the incident magnetic field.

According to White’s model, the *SE* can be explained by Equation (6) [26,27]:

(6) $$SE\;=\;168-10log\left(\frac{K_{c}f}{K\;}\right)+1.315t\sqrt{\frac{fK\;}{K_{c}}}$$

where *Kc* [S/cm] is the copper conductivity (5.82 × 105 S/cm), *f* [MHz] is the frequency, *t* [cm] is the thickness, and *K* [S/cm] is the volume conductivity of the conductive material, which can be calculated via volume electrical resistance via Equation (7):

(7) $$K\;=\;\frac{1}{\rho_{v}}$$

Equation (6) can be re-written as:

(8) $$SE\;=\;168-10log\left(K_{c}f\rho_{v}\right)+1.315t\sqrt{\frac{f\;}{\rho_{v}K_{c}}}$$

The volume electrical resistivity and *SE* at 1.5 Hz frequency for all samples are listed in Table 3. To evaluate the results of different samples, the SE obtained at 1.5 GHz frequency was used. This frequency was found to be important because it is close to the frequency used by many working devices (e.g., cell phones, GPS, and Wi-Fi routers) [26]. The results are very close to the experiment results at 1.5 GHz, especially for MEFTEX 20 and MEFTEX 30. It was clear that the experimental SE value of the single-layer MEFTEX 10 differs greatly from the theoretical value. This is the reason for providing the theoretical value even though it is not designed and derived for the ultrathin fabrics used in this work. The thickness of MEFTEX 10 is significantly less (0.042 ± 0.11 mm; cf. Table 1) than the other fabrics used in this work, which contributes to the great difference in the theoretical EM SE at 1.5 GHz (cf. Table 3).

### 3.3. Multilayer MEFTEX SE and SEG

**Figure 7:**
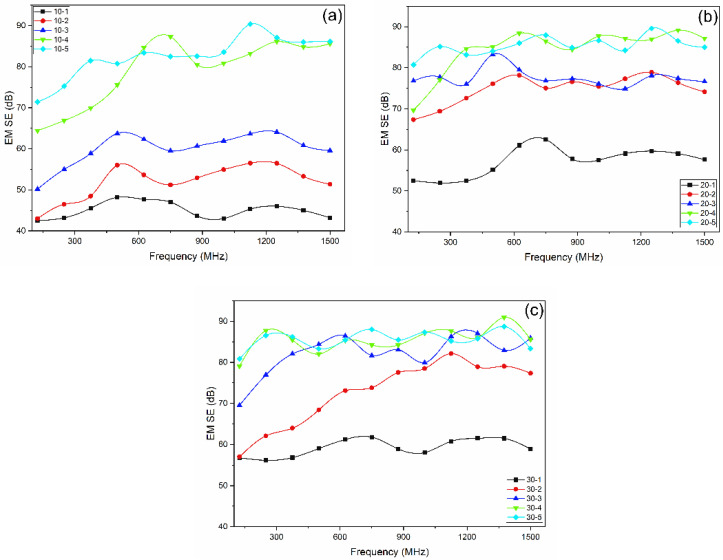
EMI shielding effectiveness (EMSE) of increasing layers for MEFTEX. (**a**) MEFTEX 10 (**b**) MEFTEX 20 (**c**) MEFTEX 30.

Adding a layer of MEFTEX caused SE to significantly increase. These results can be observed in Figure 7. Compared to single-layer MEFTEX, the multilayer MEFTEX provides significantly increased EMI shielding properties. After calculating the average SE in the frequency band from 30 MHz to 1.5 GHz, the SE of five layers of MEFTEX 10 (10-5) increased 83.3% compared to one-layer MEFTEX 10 (10-1). Five-layer MEFTEX 20 (20-5) and five-layer MEFTEX 30 (30-5) improved the electromagnetic shielding effect by 49.13% and 44.27% respectively, in comparison to one-layer MEFTEX 10. To explain this result, Equation (8) clarifies that, by increasing thickness and decreasing volume electrical resistivity, the SE can be increased. The test results reinforce this. By increasing layers, the thickness is increased and the volume electrical resistance decreases, as shown in Table 4.

For multilayer MEFTEX, the *SE* at 1.5 GHz was always above 40 dB, which is classifies it in the “Excellent” category for professional use. The maximum SE at 1.5 GHz can be reached at 87.14 dB by five layers of MEFTEX 20. This performance can be classified as an “Excellent” grade. From one layer to two layers, the SE increase rate compared to the previous layer was from 18.91% to 31.26%, two layers to 3 layers are between 3.37% and 15.98%, and from 3 layers to 4 layers is 2.67–43.64%. However, the increasing rate from the fourth layer to the fifth layer is only 0.37–2.47%.

To explain this result, the previously described Schelkunoff equation (Equation (4)) based on the transmission theory was used.

(9) $$SE_{R}\;=\;106+10lg\left(\delta_{r}/fu_{r}\right)$$

(10) $$SE_{A}\;=\;131.43t\sqrt{fu_{r}\delta_{r}}$$

where *f* is frequency [Hz], $u_{r}$ is the permeability of material relative to copper, $\delta_{r}$ is the electrical conductivity of the material relative to copper, and t is the thickness [m] [27].

For multilayer structured MEFTEX, the reflection loss $SE_{R}$ is relatively constant compared to a single layer (Equation (9)). When the number of layers is increased, the absorbing loss $SE_{A}$ increases due to the change in thickness (Equation (10)). However, the transmitted EM wave will decrease due to the decreasing porosity. When the thickness increases from the fourth layer to the fifth layer, the number of pores that electromagnetic waves can penetrate slightly reduces. Therefore, when the number of layers increases, the increase rate of the shielding effectiveness compared to the previous layer will decrease.

For different applications of EMI shielding materials, the weight of the electromagnetic shield material is significant as well. In these cases, the specific electromagnetic EMSE, *SEG* [dB·m^2^/g] can be calculated according to the following equation:

(11) $$SEG\;=\;\frac{SE}{w}$$

where *w* (g/m^2^) is the planar mass.

**Figure 8:**
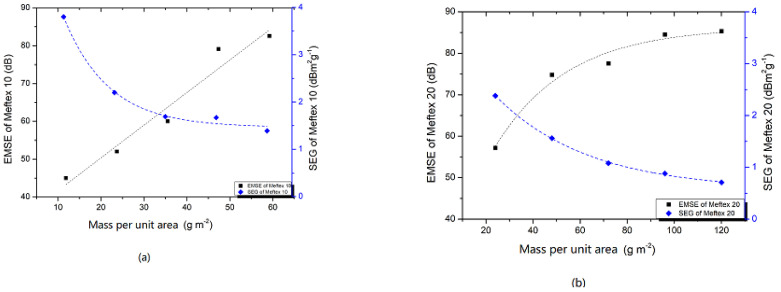

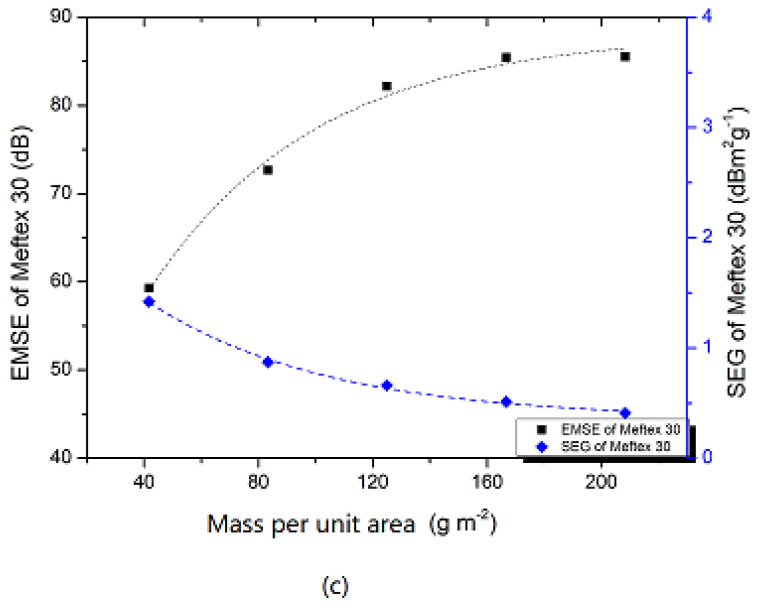
SEG value and relationship between SE and planner mass (**a**) MEFTEX 10 (**b**) MEFTEX 20 (**c**) MEFTEX 30.

The material with a higher *SEG* is desirable. It is often possible to make the shield thicker for a higher shielding ability. For the multilayer MEFTEX, the SEG decreases with the increasing of layers (see Figure 8). The SEG value for aluminum foil is 1.42 (*SE* = 78 dB, w = 55 g/m^2^), which was the best material tested in reference [28,29]. A single layer of MEFTEX with all specifications provided higher *SEG* than aluminum foil, and multilayer MEFTEX 10 with up to four layers provided higher *SEG* than 1.42. This is because of the ultra-thin characteristics and high porosity of MEFTEX 10. For the statistical approach, the *SE* and planner mass can be fitted using the exponential function, and the final fitting model had R2 > 0.9 for all three samples, which confidently proves an exponential relationship between mass per unit area and *SE*. In contrast, the increase of planner mass via layer increasing is linear. The difference of increasing trend between mass per unit area and *SE* causes *SEG* to decrease as layers are added.

### 3.4. Air Permeability Is Influenced by the Multilayer Structure

**Figure 9:**
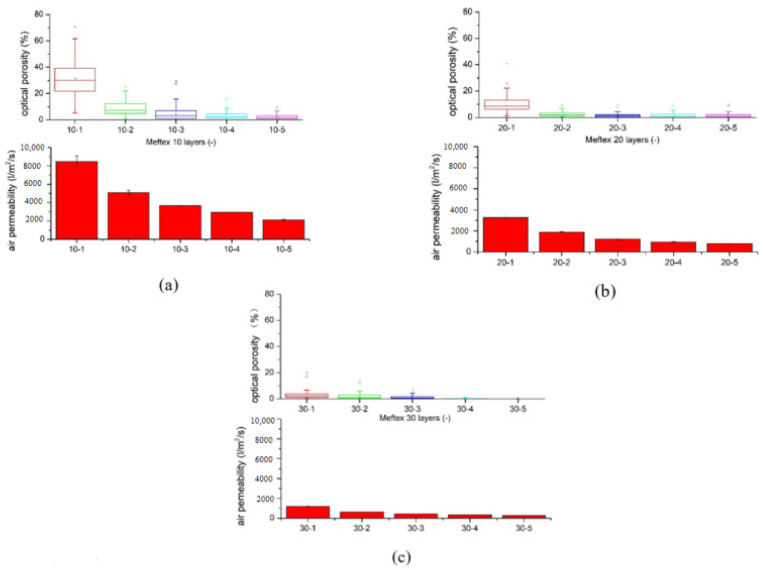
Optical porosity presented by porous distribution boxplot and air permeability influenced by increasing of layers (**a**) MEFTEX 10 (**b**) MEFTEX 20 (**c**) MEFTEX 30.

As the number of layers increases, the porosity of the nonwoven structured sample decreased. Using the MATLAB^®^ image analysis program, the optical porosity influenced by the layer layout is presented via the distribution of porosity in boxplot Figure 9, which also proves the aforementioned conclusion. The total volume of void space within a specified area of the MEFTEX 30 fabric is smaller than the other fabrics. For this reason, MEFTEX 30 has the lowest porosity (cf. Figure 4a–c).

As we know, porosity has a significant influence on the air permeability of textile material. The diameter of pores influences the air permeability of EMI shielding textiles and affects their SE. In the case of metal foil, the SE can be calculated by the following empirical equation (Equation (12)) [28]:

(12) $$SE\;=\;20lg\left(\frac{1}{T_{t}+T_{h}}\right)\;=\;20lg\left(\frac{1}{T_{t}+4n{\left(q/F\right)}^{\frac{3}{2}}}\right)$$

*T_t_* is the transmission coefficient of the total shielding metal, *T_h_* is the transmission coefficient of the pores on the shielding metal foil, *n* is the number of pores, *q* is the area of each pore, and *F* is the total area of the shielding metal foil.

**Figure 10:**
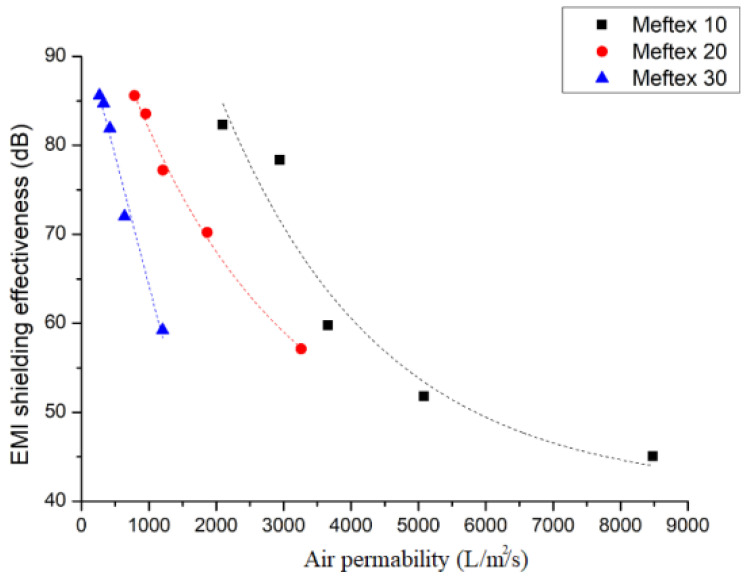
The relationship between air permeability and EMSE of MEFTEX.

According to this equation, if the metal foil pores are getting larger, the SE becomes lower. This conclusion may indicate a contradiction for air permeability and SE. To illustrate this problem, the relationship of the air permeability of multilayered MEFTEX and its SE (average SE from frequency 30 MHz to 1.5 GHz) is presented in Figure 10. All three groups of MEFTEX indicate that with increasing air permeability, the SE decreases, creating an exponential relationship (R2 > 0.9). The increase in air permeability can be seen as a decrease in sample thickness. By combining the conclusions from Equations (8) and (10), it can be inferred that when the number of layers is increased, the sample’s porosity will decrease. These two factors can increase SE.

There is one point that should be noticed. Three layers of MEFTEX 10 have a similar thickness, mass per unit area (cf. Table 1), and volume resistivity (cf. Table 3) compared to one-layer MEFTEX 30. The same principle applies when comparing two layers of MEFTEX to one layer of MEFTEX 20, indicating a similar EMI shielding property. The reason is that, for the same material, increasing the number of layers at the same time causes the volume resistivity will decrease. When the thickness and resistivity are similar, the SE performance should not be significantly different, which has been proven from the SE test result. (cf. Figure 7) However, the air permeability of three-layer MEFTEX 10 is much better than one-layer MEFTEX 30. The same applies when comparing two layers of MEFTEX 10 to one-layer MEFTEX 20 and four layers of MEFTEX 10 to two layers of MEFTEX 20. The difference in air permeability is caused by the difference in porosity, which can be observed via optical porosity. The optical porosity of 3 layers of MEFTEX 10 is higher than one-layer MEFTEX 30. The mass per unit area and thickness of one layer of MEFTEX 30 and three layers of MEFTEX 10 are similar, but the fiber arrangement of MEFTEX 10 and MEFTEX 30 can vary. It cannot simply be interpreted that the porosity of three layers of MEFTEX 10 is similar to one layer of MEFTEX 30, but it can be inferred from this research.

One advantage of copper-coated material is combining the suitable EMI shielding property and textile wearable properties, including air permeability. In this study, a higher mass per unit area fabric (MEFTEX 30) provided better SE. However, on the same SE level, the lower mass per unit area fabric (MEFTEX 10) shows better air permeability. Considering the balance of SE and air permeability for MEFTEX (Figure 10), three to four layers of MEFTEX 10 will perform average SE of 59.79 dB–78.38 dB in the frequency band 30 MHz to 1.5 GHz, and average air permeability of 2942 L/m^2^/s–3658L/m^2^/s [30].

## 4. Conclusions

In this research, three different mass per unit area EMI shielding materials, MEFTEX 10, MEFTEX 20, MEFTEX 30, were studied to determine whether multilayered structure influences SE and air permeability. Observed by SEM and EDX, a uniform copper coating is plated on the surface of the polyester fiber, which gives the material excellent electromagnetic shielding performance and maintains the porosity of the base material itself. MEFTEX 30 has the best SE effect among single-layer materials, which is around 58.92 dB, because of its higher thickness and lower volume electrical resistivity. According to the shielding principle, a thicker material increases the absorption attenuation capacity of the shield under the same shielding material. In this research, comparing the SE with two materials of similar thicknesses, two layers of MEFTEX 10 and one layer of MEFTEX 20, as well as three layers of MEFTEX 10 and one layer of MEFTEX 30, there is no significant difference in SE performance. As the number of material layers increases, the shielding effect of five layers of MEFTEX is significantly higher (44.27–83.8%) than one-layer MEFTEX. After the number of layers is increased, the porosity is significantly reduced, and the air permeability is also reduced. Nevertheless, within a considerable EMI shielding range around 59.79 d–78.38 dB, the air permeability of three to four layers of MEFTEX 10 material was maintained from 2942 L/m^2^/s–3658 L/m^2^/s.

## Figures and Tables

**Table 1:** Characteristics of the samples.

Sample Number	Sample Details	Thickness (1 Layer) (mm)	Mass Per Unit Area (1 Layer) (g/m^2^)	Deposit of Cu Per Unit Area (g/m^2^)
MEFTEX 10	100% PET nonwoven	0.042 ± 0.11	11.84	1.84
MEFTEX 20	100% PET nonwoven	0.074 ± 0.008	24.01	4.01
MEFTEX 30	100% PET nonwoven	0.112 ± 0.01	41.67	11.67

**Table 2:** EDX analysis of MEFTEX surface.

Sample Number	Element	Wt [%]	Standard Deviation	Element	Wt [%]	Standard Deviation
Meftex 10	Cu	75.87	2.12	C	16.32	1.03
	O	7.28	0.87	Ti	0.36	0.08
	Ca	0.16	0.32			
Meftex 20	Cu	45.2	1.21	C	33.49	1.09
	O	20.18	0.38	Ti	0.47	0.04
	Ca	0.67	0.15			
Meftex 30	Cu	63.52	2.01	C	23.94	0.9
	O	11.94	1.08	Ti	0.35	0.02
	Ca	0.24	0.14			

**Table 3:** Volume electrical resistivity and EMSE, SE_R_, SE_A_, SE_M_ at 1.5 GHz.

Sample Number	Thickness [mm]	Volume Electrical Resistivity [Ω·mm]	The Experiment Result of EM SE on 1.5 GHz [dB]	SE_R_ [dB]	SE_A_ [dB]	SE_M_ [dB]	The Theoretical Calculated Result of EM SE on 1.5 GHz [dB]
MEFTEX 10	0.042	5022.78	45.2	36.79	8.41	−1.47	51.58
MEFTEX 20	0.074	1676.40	57.64	44.71	12.93	-	56.34
MEFTEX 30	0.112	991.50	58.91	43.76	15.14	-	58.62

**Table 4:** Volume electrical resistivity change and thickness change with increasing of layers.

Sample Code *	Volume Electrical Resistivity [Ω·mm]	Sample Code *	Volume Electrical Resistivity [Ω·mm]	Sample Code *	Volume Electrical Resistivity [Ω·mm]
10-1	5022.78	20-1	1676.40	30-1	991.50
10-2	1640.93	20-2	663.40	30-2	430.43
10-3	963.80	20-3	433.67	30-3	297.05
10-4	126.70	20-4	325.73	30-4	248.19
10-5	60.04	20-5	249.12	30-5	204.11

* 10-1: 1 layer of MEFTEX 10, 10-2: 2 layers of MEFTEX 10. Same rule for other samples.

## Data Availability

Not applicable.

## Appendix A

**Table A1:** Classification of EM SE values on textiles for general use [29].

Type	Grade	Shielding Effectiveness (dB)	Classification	Percentage of Electromagnetic Shielding (%)
Class I Professional use	AAAAA	SE > 60 dB	Excellent	ES > 99.9999%
	AAAA	60 dB ≥ SE > 50 dB	Very good	99.9999% ≥ ES > 99.999%
	AAA	50 dB ≥ SE > 40 dB	Good	99.999% ≥ ES > 99.99%
	AA	40 dB ≥ SE > 30 dB	Moderate	99.99% ≥ ES > 99.9%
	A	30 dB ≥ SE > 20 dB	Fair	99.9% ≥ ES > 99.0%
Class II General use	AAAAA	SE > 30 dB	Excellent	ES > 99.9%
